# Metabonomics profile analysis in inflammation-induced preterm birth and the potential role of metabolites in regulating premature cervical ripening

**DOI:** 10.1186/s12958-022-01008-y

**Published:** 2022-09-06

**Authors:** Yan Yan, Zhuorong Gu, Baihe Li, Xirong Guo, Zhongxiao Zhang, Runjie Zhang, Zheng Bian, Jin Qiu

**Affiliations:** 1 Obstetrics and Gynecology Department, Tongren Hospital, Shanghai Jiao Tong University School of Medicine, No.1111, XianXia Road, Shanghai, 200336 China; 2 Hongqiao International Institute of Medicine Tongren Hospital, Shanghai Jiao Tong University School of Medicine, No.1111, XianXia Road, Shanghai, 200336 China

**Keywords:** Preterm birth, Premature cervical ripening, Metabonomics, Taurine, Cervical smooth muscle cells

## Abstract

**Background:**

Preterm birth (PTB) is the primary cause of infant morbidity and mortality. Moreover, previous studies have established that PTB is related to premature cervical ripening. However, the underlying mechanism remains to be elucidated. This study sought to identify differentially expressed metabolites and investigate their potential biological functions in PTB.

**Methods:**

Pregnant C57BL/6 J mice were treated with either LPS or normal saline and cervical alterations before labor were detected by staining. Metabolic profiles in the plasma of PTB and control mice were examined through non-targeted metabonomics analyses, quantitative polymerase chain reaction and immunofluorescence staining were performed on human cervical smooth cells.

**Results:**

The study demonstrated that the mRNA and protein levels of α-SMA, SM-22, and calponin in cervical smooth muscle cells of PTB mice were lower while OR was higher at both mRNA and protein levels compared to the CTL group. A total of 181 differentially expressed metabolites were analyzed, among them, 96 were upregulated, while 85 were downregulated in the PTB group. Differentially expressed metabolites may play a role in STAT3, RhoA, mTOR, TGF-β, and NK-κB signaling pathways. Furthermore, when treated with taurine, the levels of α-SMA and SM-22 in human cervical smooth muscle cells were elevated, whereas that of connexin-43 was decreased.

**Conclusion:**

Our study highlighted the changes of metabolites in the peripheral blood changed prior to PTB and revealed that these differentially expressed metabolites might participate in the development of premature cervical ripening. Taurine was identified as an important metabolite may modulate human cervical smooth muscle cells. Our study provided new insights into the mechanism underlying premature cervical ripening in PTB.

**Supplementary Information:**

The online version contains supplementary material available at 10.1186/s12958-022-01008-y.

## Background

Preterm birth (PTB) is defined as the delivery of a baby before the 37^th^ week of gestation [1]. It occurs in 8%-13% of all pregnancies and is a major cause of perinatal mortality and morbidity. It also brings a considerable economic burden to families and society [2]. As many as 50% of spontaneous PTBs are linked to inflammatory cascades that induce uterine contractions. As expected, certain subclinical infections are challenging to diagnose [3]. Although perinatal medical technology has greatly improved, with routine treatments such as antibiotics, vaginal progesterone, tocolytic agents, cervical pessary, and cervical cerclage being widely used, the side effects of drugs and operative complications limit their clinical applications and have not effectively reduced the incidence of PTB [4].

Cervical maturation is characterized by cervical length shortening and is a prerequisite for delivery. It is usually accompanied by changes in the cervical smooth muscle cells (CSMC) and extracellular matrix. Thus, premature cervical remodeling could lead to spontaneous PTB [5]. There may be a specialized sphincter in the internal os and CSMC, playing a key role in the propagation of uterine contractions and cervical remodeling [6]. The dynamic changes in CSMC run through the entire labor process [7], whereby soft cervical extracellular matrix may lead to decreased CSMC contractile tone and cervical length (supplementary figure). Moreover, the predisposition to sphincter laxity contributes to PTB [8]. Endocrine abnormalities, immune-mediated inflammatory reactions, abnormal gap junction of cervical myocytes, and changes in calcium channels caused by various factors may also induce cervical maturation [9–11]. However, as a key phase during labor, the mechanism initiating CSMC changes in PTB remains unclear.

Metabonomics describes dynamic changes and provides quantitative and qualitative information on the metabolites present in biological systems [12]. Further advancements in metabolic techniques have enabled the identification of low abundance metabolites and the research of complex biological samples [13]. For instance, metabonomics was used for screening neonatal phenylketonuria and congenital hypothyroidism [14]. Another study reported that 53 serum metabolites could potentially improve the risk assessment of some adverse maternal health conditions (e.g., gestational diabetes and preeclampsia) [15, 16]. Regarding PTB biomarkers, metabolites found in maternal peripheral blood following full-term birth and PTB are distinct, with some of them affected by gestational age [17]. Not only do active metabolites serve as early warning signs of diseases, but they also have biological functions. Formate, methanol, acetone in the vaginal fluid, retinyl palmitate, At-Retinal, 13-cis-Retinoic acid, and folate in the blood significantly correlate with inflammation, thereby facilitating PTB cascades [18]. Although current lines of evidence also indicate that the metabolite modulate various metabolic mechanisms and inflammatory processes, including PTB [19], the role of active metabolites in premature cervical maturation and PTB has not been reported yet.

Lipopolysaccharide (LPS) is a plasma membrane component found in numerous microorganisms and is associated with acute chorioamnionitis. Indeed, LPS-induced inflammation leads to PTB [20]. Herein, the changes of metabolites and cervical ripening processes in PTB were explored in LPS-induced PTB mice. Non-targeted metabonomics methods (liquid chromatography-mass spectrometry, LC–MS) were employed to analyze serum samples from mice and identify the differentially expressed metabolites of inflammation-induced PTB. It was noted that the differentially expressed metabolites might regulate cervical ripening. Additionally, when treated with taurine, the level of CSMC markers and contraction-associated proteins changed. This study may provide a new perspective on the mechanism of premature cervical ripenings in PTB.

## Methods

### Animal protocols

Ten to eleven weeks old C57BL/6 mice were purchased from the experimental animal center of the Minhang campus of East China Normal University. The animals were exposed to a 12 h dark/12 h light cycle at a fixed humidity level (50–60%) and temperature (21 ± 2 °C). The mice were caged according to a female to male ratio of 2:1, while pregnancy confirmation was determined by obvious visual inspection of the vaginal plug and was marked as day 0 of pregnancy (Day 0). Afterward, the pregnant mice were randomly assigned into 2 groups: intraperitoneal injection of PBS group (control group) and intraperitoneal injection of LPS (PTB group). At 15.5 days of gestation, pregnant mice were intraperitoneally injected with LPS (ultrapure-LPS, Dakewe Biotech Co. Ltd. USA) at a dose of 50 μg/ kg. Following the first PBS or LPS injection, the mice were monitored hourly for any signs of labor (decreased movement, vaginal bleeding, and preterm delivery). The beginning of preterm delivery was defined as the delivery of the first pup. The mice started giving birth 10–18 h post-injection (mainly between 10–12 h). At the initiation of labor in PTB mice, all mice were sacrificed, then blood samples and cervical tissues were collected. All mice were at the same gestational age. The animal study was reviewed and approved by The Ethics Committee of Animal Experiments at Shanghai Tongren Hospital, Shanghai Jiao Tong University School of Medicine.

### Staining

After labeling the cervical tissues, the specimens were cut into serial macroscopic slices. Some cervical tissues were stained with hematoxylin and eosin (H&E), and the remaining were used for immunohistochemical staining. The slides were observed under a light microscope for cellular changes, and photographs were taken digitally with a NanoZoomer S60 (Hamamatsu, Japan).

H&E staining: The samples were fixed in 10% formalin saline for 24 h, then transferred into 70% ethanol and processed to paraffin-embedded blocks to produce 5 μm thick sections. The samples were then deparaffinized in xylene, rehydrated in descending concentrations of alcohol, and lastly stained with hematoxylin and eosin.

Immunohistochemical staining: Following dehydration in graded ethanol and transfer to xylene, the tissues were embedded in paraffin. After the slices were microwaved in a citrate–phosphate buffer (pH 6.0) to retrieve antigens, they were treated with 3% hydrogen peroxide followed by 10% normal goat serum blocking at room temperature for 30 min. Next, the slices were incubated with primary antibodies (α-smooth muscle actin [α-SMA], smooth muscle heavy chain 22 [SM-22], calponin, cyclo-oxygenase 2 [COX-2], oxytocin receptor [OR], and connexin-43) diluted in PBS for 24 h at 4 °C. The sections were then incubated with secondary antibodies for 30 min at room temperature following several washes in PBS. Finally, the signals were detected using a biotin-streptomycin-hydroxide system using diamorphine as the chromosome. Negative controls were performed with the primary antibodies replaced by PBS.

Immunofluorescence staining: The CSMC were cultured on slides and treated with metabolites, then fixed in 4% paraformaldehyde. The sections were rinsed in PBS and incubated in a non-immune blocking solution for 2 h at room temperature (Triton 100 × and bovine serum protein were dissolved in PBS). Incubation with primary antibodies was performed for 18 h at 4℃. Then, the sections were washed with PBS and incubated with a secondary antibody for 1 h at room temperature way from light. Finally, the sections were again rinsed with PBS, mounted with fluoroshield and DAPI mounting medium (Cat# ab104139, Abcam), and photographed with a Confocal Laser Scanning Microscope (Leica, Germany).

The information of the primary antibodies was as follows: α-SMA polyclonal antibody (Cat No.14395–1-AP, Proteintech, USA), SM22 polyclonal antibody (Cat No.10493–1-AP, Proteintech, USA), calponin polyclonal antibody (Cat No. 13938–1-AP, Proteintech, USA), COX-2 monoclonal antibody (Cat No. 66351–1-Ig, Proteintech, USA), connexin-43 polyclonal antibody (Cat No. 26980–1-AP, Proteintech, USA), and OR polyclonal Antibody (Cat No. 23045–1-AP, Proteintech, USA).

The information of the secondary antibodies was as follows: CoraLite488-conjugated Goat Anti-Mouse IgG(H + L) (Cat No.SA00013-1, Proteintech, USA) and CoraLite488-conjugated Goat Anti-Rabbit IgG(H + L) (Cat No.SA00013-2, Proteintech, USA).

### Cell culture

This study was approved by the Ethics Committee of Shanghai Tongren Hospital, Shanghai Jiao Tong University School of Medicine (No.2020–035-02). Cervical tissues were obtained from women undergoing a total hysterectomy for benign indications. The cervical tissues were quickly passed through 75% alcohol and washed twice with PBS, then placed in a 6 cm dish and chopped as much as possible. The chopped tissues were digested using 200 U/mL collagenases (Invitrogen, USA) and 25 kU/mL trypsin (Invitrogen, USA) for 2 h under 37℃ conditions. After observing under the microscope to confirm the tissues became loose, the large tissue pieces were filtered out with a 100-mesh cell sieve (BD, USA)and transferred to a 15 ml centrifuge tube centrifuged at 1500 rpm for 5 min before discarding the supernatant. Afterwards, 4 ml of culture base was resuspended before dividing into Petri dishes. The resuspended culture base was maintained in DMEM (Gibco, USA) with 10% FBS (Gibco, USA), 100 U/ml penicillin, and 100 μg/ml streptomycin in a humidified atmosphere at 37 °C with 5% CO2. The growth medium was changed every 2–3 days. In order to maintain the viability and phenotype of primary muscle cells, continuous plating was carried out, and the culture was limited to 5 generations.

### Quantitative polymerase chain reaction (qPCR)

RNA from tissues and cells was extracted using the Trizol reagent (Invitrogen, USA). Then, total RNA was converted into complementary DNA (cDNA) using a Reverse Transcription Kit (Invitrogen, USA). The qPCR was carried out using SYBR Green qPCR SuperMix (Invitrogen, USA) and the ABI PRISM 8000 Sequence Detection System according to the manufacturer’s protocols. The PCR primers are depicted in Table 1. The following conditions were used for amplification: 50℃ for 2 min, 95℃ for 2 min, 40 cycles of 95℃ for 15 s and 60℃ for 60 s. The fluorescence intensities of the probes were plotted against the PCR cycle numbers. GAPDH served as a control gene for gene expression normalization using the 2^–ΔΔCt^ method.

**Table 1:** List of primers

Gene	Sequence	Sequence(3'-5')
a-SMA	F	AGCCATCTTTCATTGGGATGG
a-SMA	R	CCCCTGACAGGACGTTGTTA
SM-22	F	CGGCCTTTAAACCCCTCACC
SM-22	R	CATGTTGAGGCAGAGAAGGCT
calponin	F	CTGTTGCGCTTGTCTGTGTC
calponin	R	TTTCTGGGCCAGCTTGTTCT
COX-2	F	CATTGGGGGAAAGGCGTGA
COX-2	R	CCATGTCTGGGCACCTCTCTTT
oxytocin receptor	F	GGCCGTGTTCCAGGTTCTC
oxytocin receptor	R	TGCAAGTATTTGACCAGACGAC
connexin-43	F	CATTGGGGGAAAGGCGTGA
connexin-43	R	CCATGTCTGGGCACCTCTCTTT

*F* represents forward primer, *R* represents reverse primer

### Blood sample preparation

At the onset of PTB mice, all mice were sacrificed, blood samples were collected. Venous blood (1 ml per mouse) was drawn from the tail vein of mice. The blood was centrifuged at 3500 rpm for 10 min at 4℃. Next, the supernatant was transferred to a 1.5 mL centrifuge tube as a serum sample for LC–MS detection. Each frozen serum sample was thawed separately at room temperature. Then, 100 µL serum was added to a 300 µL methanol solution containing 5 µg/ mL L-2-chlorophenylalanine as the internal standard and rotated for 2 min. Centrifugation was done at 13,000 rpm at 4℃ for 10 min. Finally, 200 µL supernatant was extracted. The same volume of serum was extracted from all samples and evenly mixed to prepare quality control (QC) samples.

### Detection of metabolic profiling by LC–MS

Ultra-High Performance Liquid chromatography (Ultimate 3000, USA) combined with the thermo-Orbitrap Elite mass spectrometer was utilized for the LC–MS analysis. The system was equipped with an electrospray ionizationsource and operated in either positive or negative ionizationmode using a mass resolution of 70, 000 at an m/z of 200. Data-dependent (dd-MS2, Top *N* = 10) MS/MS mode with a full scanmass resolution of 17, 500 at an m/z of 200 was used. The scanrange was 100–1, 500. Metabolic profiles in electrospray ionization (ESI) positive and ESI negative ion modes were performed using an ACQUITY UPLC I-Class system (Waters Corporation, USA) coupled with an AB SCIEX Triple TOF 5600 System (AB SCIEX, USA). The binary gradient elution systems consisted of water containing 0.1% formic acid, v/v (A), and acetonitrile containing 0.1% formic acid, v/v, (B). 20% B for 2 min; 60% B for 4 min; 100% B for 11 min; 100% B for 13 min % B for 13.5 min and finally, 5% B for 14.5 minutesnthe above steps, separation was achieved. The chromatographic conditions were as follows: injection volume was 2 μl; column temperature was 25 °C; flowrate was 0.35 ml/min. Data were acquired in centroid mode using the Thermo Excalibur 2.2 software (Thermo Fisher Scientific, USA).

### Statistical analysis

Statistical analyses were performed by the SPSS Statistics 22.0 software (IBM, USA) and GraphPad Prism 9.0 software (GraphPad Software Inc, USA). Statistical significance was determined according to the sample distribution and homogeneity of variance, while statistical comparisons between two groups were determined by the t-test. The metabolic data were acquired using the Thermo Xcalibur 2.2 software (Thermo Scientific, USA). Peak alignment and extraction were performed using the Compound Discoverer software (Thermo Scientific, USA). Next, a data table containing information regarding the retention time, m/z, and peak area was obtained. The edited data matrix was imported into the SIMCA-P 11.0 software (Umetrics, Sweden) for multivariate statistical analysis, principal components analysis (PCA), and partial least squares discrimination analysis (PLS-DA). The unsupervised PCA analysis assessed the overall trend of segregation between the samples, while a supervised PLS-DA analysis model screened for significantly different metabolites between the PTB and control groups. The ion peaks were normalized and Pareto-scaled. According to PLS-DA model, the variables with variable importance in the projection (VIP) value > 1.0 were selected and p < 0.05 was considered as statistically significant. Bonferroni correction was used for multiple testing adjustment. In order to identify these potential biomarkers, the accurate ion mass was input into the human metabolome database (HMDB, https://hmdb.ca) databases to match the exact molecular weight, and MS1/MS2 fragment ions were automatically searched. Finally, in order to confirm the structure of the compound, we used our internal standard metabolite library, matching the exact mass,fragment ion mass, and retention time.The ingenuity pathway analysis (IPA) from the Kyoto Encyclopedia of Genes and Genomes (KEGG) online database was applied to understand the functions and interactions of genes and metabolites. * *p* < 0.05, ** *p* < 0.01 and *** *p* < 0.001.

## Results

### Characteristics of PTB mice

**Fig. 1:**
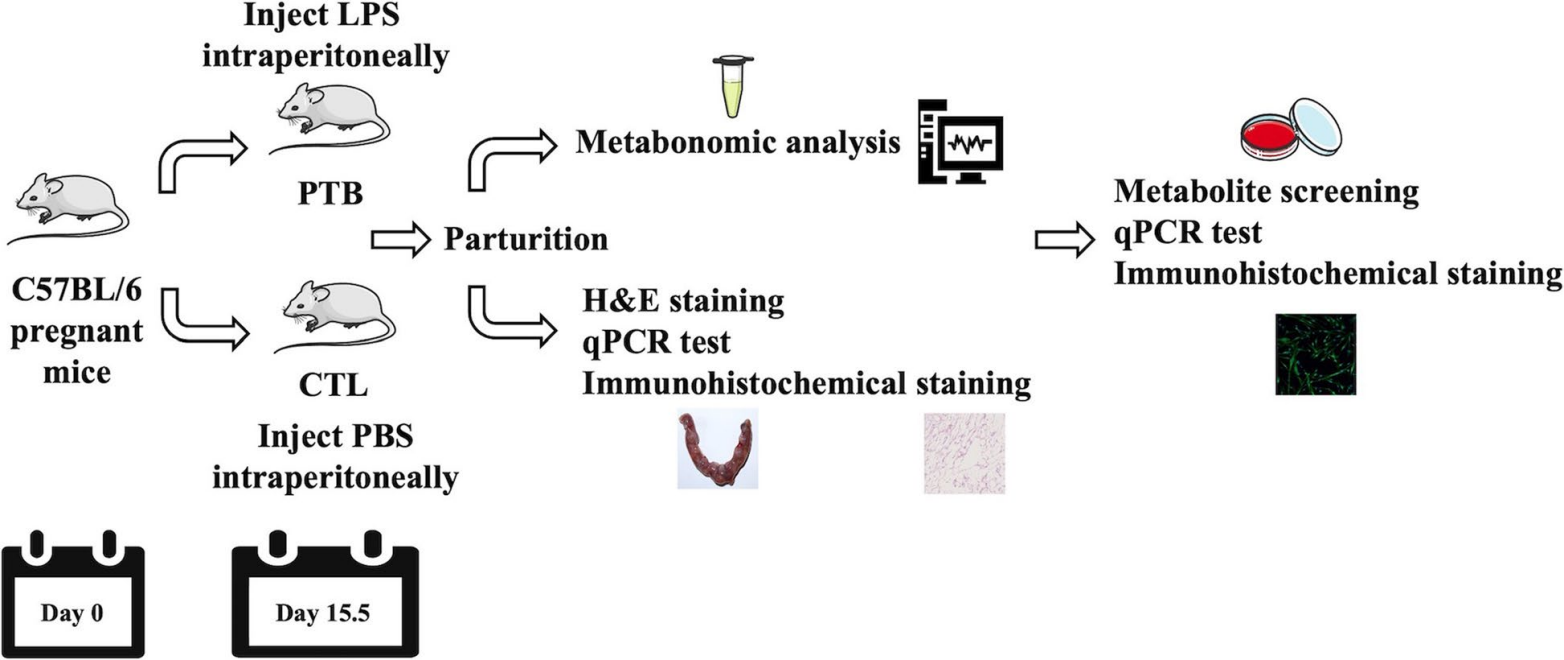
Flow chart of the research. The figure illustrates the experimental protocol. Pregnant C57BL/6 mice were treated with LPS or PBS on the 15.5th day. Most mice in the PTB group delivered during the night of day 16. Once the mice exhibited signs of premature delivery, blood and tissue samples were taken. Then, metabonomics analysis, staining, and qPCR were performed. After screening the differentially expressed metabolites, human CSMC were used to explore the potential functions of metabolites

To locate the differentially expressed metabolites, LPS was utilized to establish inflammation-induced PTB models. The experiment design is illustrated in Fig. 1. Pregnant C57BL/6 mice were randomly injected with LPS or PBS on the 15.5th day. The labor process was observed, and LPS mice gave birth 10–18 h post-injection (mostly between 10–12 h). Once the mice showed signs of premature delivery, blood and tissue samples were collected. Then, metabonomics analysis, staining, and qPCR were performed. After screening the differentially expressed metabolites, human CSMC were used to investigate the potential functions of those metabolites.

### Changes of cervical tissue in PTB mice

**Fig. 2:**
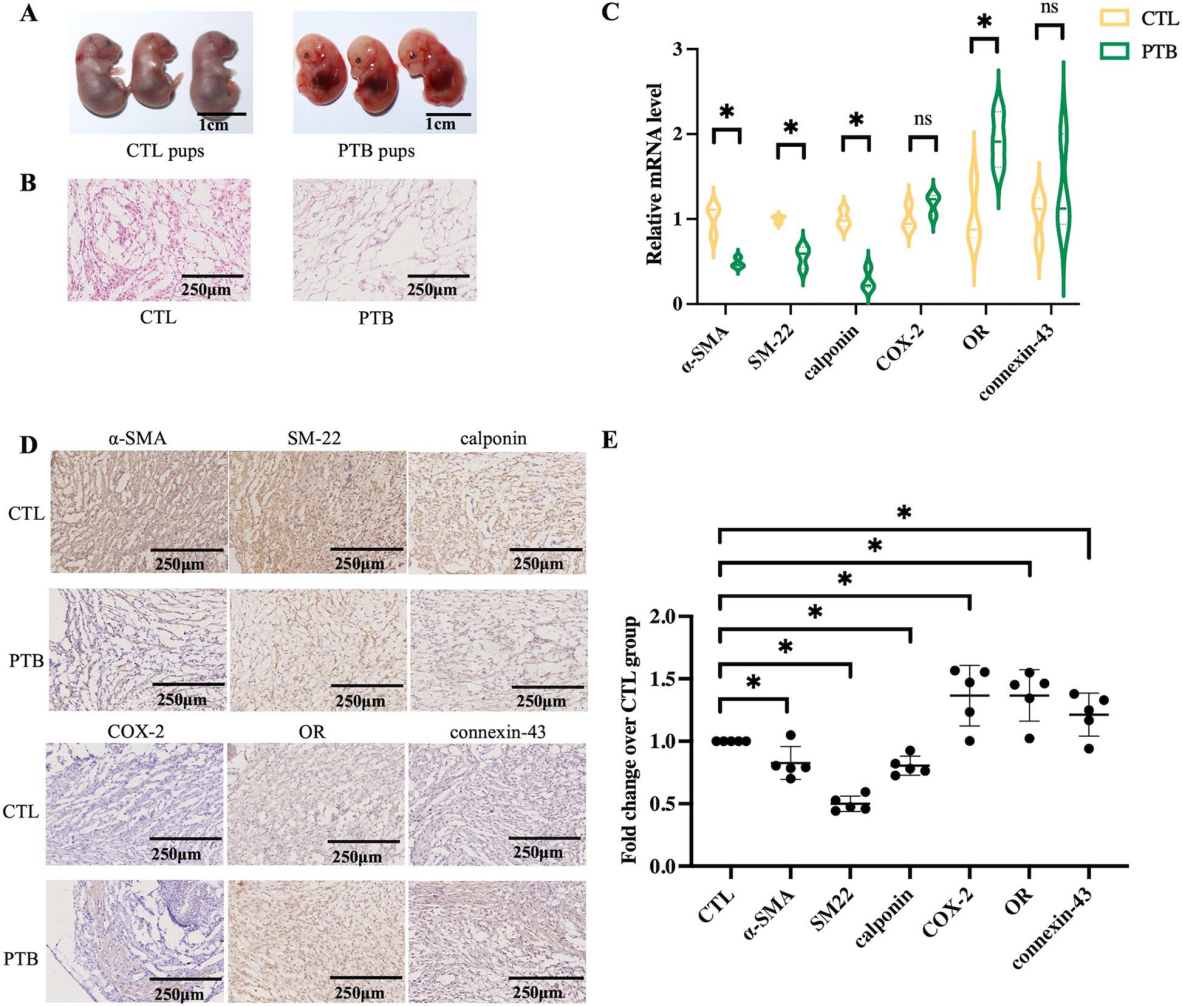
Characteristics of PTB mice. **A** The images display that the pups of PTB mice were immature. **B** Results of cervical H&E staining of the CTL and PTB groups. Histological slices revealed that there were more CSMC in the CTL group. **C** CSMC markers and uterine activation protein content in PTB mice changed. PTB mice had a higher level of OR mRNA, the relative mRNA expression of α-SMA, SM-22, and calponin was lower in the PTB group (* *p* < 0.05). **D** Immunohistochemistry was used to stain cervical tissues. The slides show that CTL mice cervix contained a significant amount of α-SMA, SM22, and calponin. The content of COX-2, OR, and connexin-43 was higher in PTB mice. **E** The semi-quantitative analysis of immunohistochemistry slides show that compared with CTL group, the content of α-SMA, SM-22, and calponin were lower, while COX-2,OR and connexin-43 were higher in PTB group (all the differences were statistically significant) (* *P* < 0.05, *n* = 5)

The pups from the PTB group were immature, signifying that the PTB mouse model was successfully established (Fig. 2A). Then, histological changes in the cervix of the mice were observed. As displayed in Fig. 2B, H&E staining of cervical slices revealed that there were more CSMC in the CTL group. Moreover, the relative mRNA expression levels in cervical tissues of mice changed as well. Compared with the CTL group, OR mRNA level in the PTB group increased significantly. Conversely, the relative mRNA expression of α-SMA, SM-22, and calponin was significantly lower in the PTB group (Fig. 2C). Next, the tissues were stained for α-SMA, SM-22, COX-2, OR and connexin-43. Immunohistochemistry identified significant levels of α-SMA, SM-22, and calponin in CTL mice, while the content of COX-2, OR, and connexin-43 were higher in PTB mice (Fig. 2D). The semi-quantitative analysis of immunohistochemistry slides show that compared with CTL group, the content of α-SMA, SM-22, and calponin were lower, while COX-2,OR and connexin-43 were higher in PTB group (all the differences were statistically significant) (Fig. 2E).

### Detection of the metabolic profile in CTL and PTB mice

**Fig. 3:**
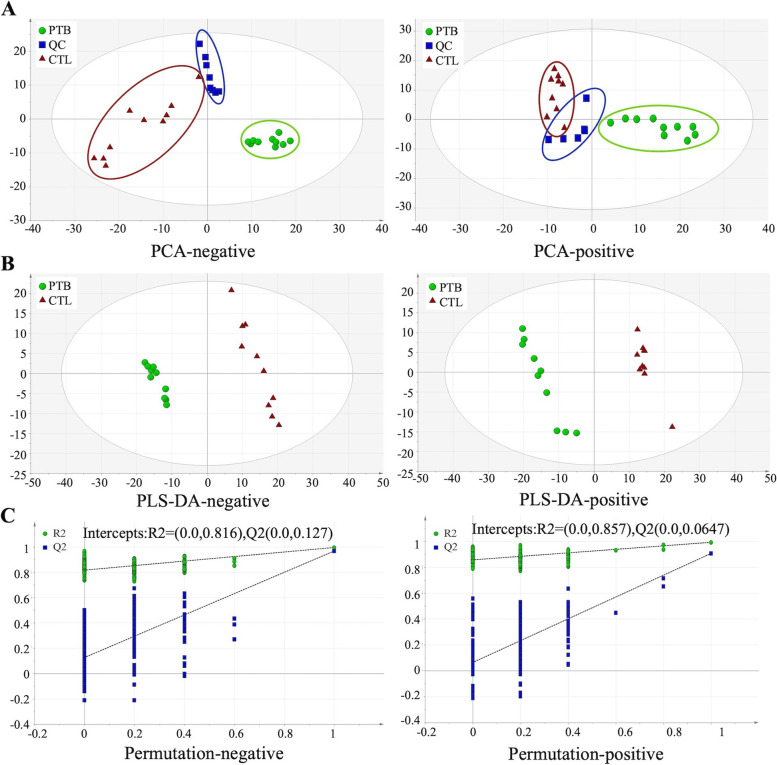
Identified metabolic profile in CTL and PTB mice. **A** Principal component analysis (PCA) showed a distinct metabolic profile in the PTB group compared to the CTL group. X-axis and Y-axis represent the first and second principal components, respectively. **B** Statistical validation with perripening analysis of the corresponding OPLS-DA model of the PTB and CTL groups. **C** Perripening tests were obtained from LC–MS data of the PTB and CTL groups. The intercept values of the regression line and the Y-axis are R2 and Q2

To examine the metabolic profile of the CTL and PTB groups, the peripheral blood of mice was taken for metabonomics analyses. The results of the PCA analysis revealed the trend of intra-group aggregation and inter-group segregation in the CTL and PTB groups (Fig. 3A). The PLS-DA model was established and determined that the PTB group’s plots were far from those of the CTL group, indicating significant differences in metabolites between the two groups (Fig. 3B). Besides, a perripening examination of the PLS-DA model was conducted. The negative R2 and Q2 intercept values between the CTL and PTB groups were (0.0, 0.816) and (0.0, 0.127), while the positive R2 and Q2 intercept values were (0.0, 0.857) and (0.0, 0.0647), respectively (Fig. 3C).

**Fig. 4:**
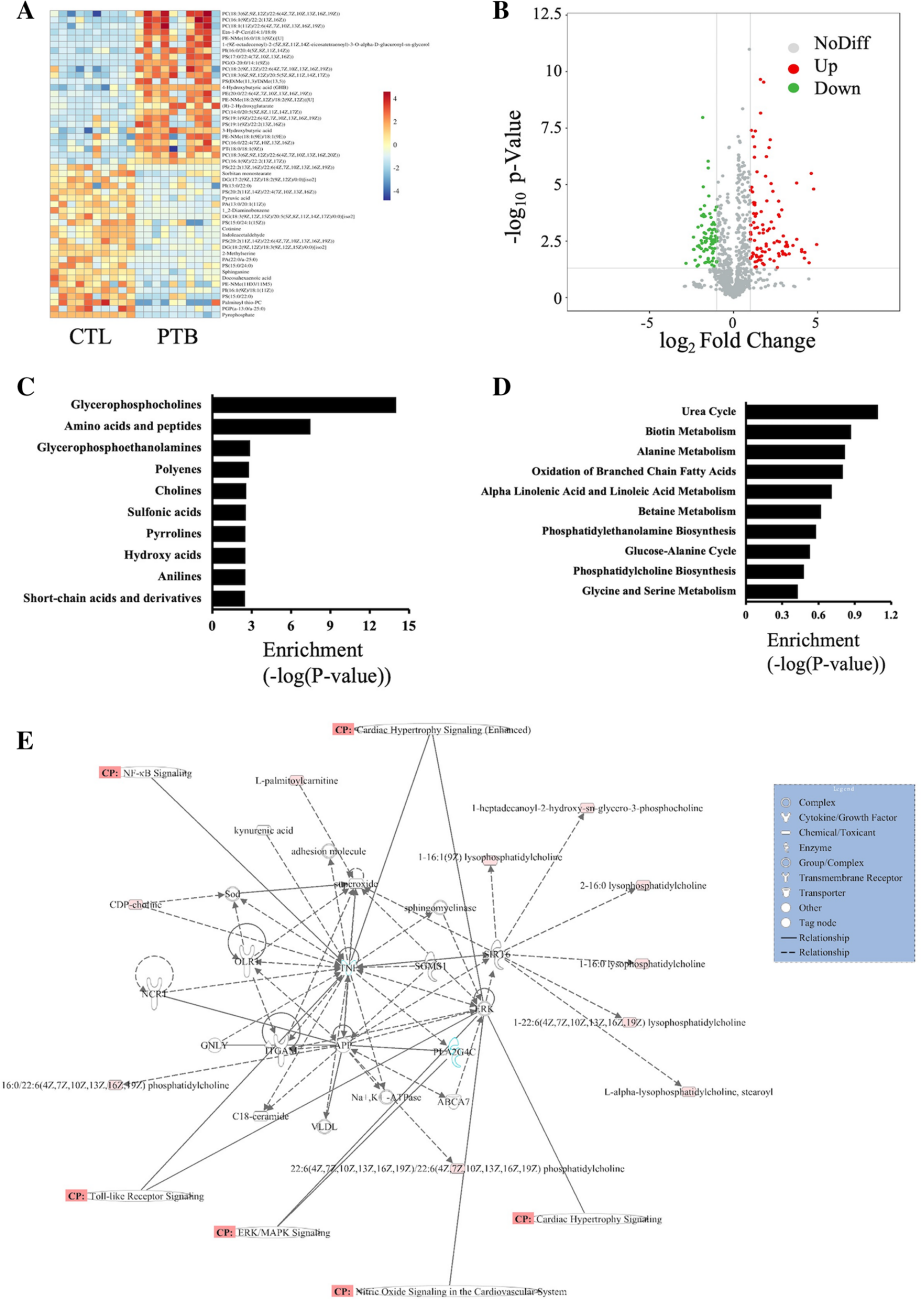
Differentially expressed metabolites in PTB mice. **A** Heatmap of 50 differential metabolites determined by LC–MS. Hierarchical clustering analysis was used to assess significantly regulated metabolites between CTL and PTB groups. Increased and decreased metabolites are depicted by red and blue, respectively. **B** The volcano map of all metabolites expressed in CTL and PTB groups. **C** The differential metabolites could be roughly categorized into several classes. **D** Enriched metabolic pathway clustering analysis. **E** IPA of metabolites related to biological network, pathways, and functions. The red nodes represent upregulated metabolites, while the green nodes represent downregulated ones. CP represents the signaling pathway related to the changed metabolites

Enrichment analyses on differential metabolites were performed. A heat map of the 50 highest differential metabolites determined by LC–MS is displayed in Fig. 4A. Hierarchical clustering analysis was used to assess significantly regulated metabolites between the CTL and PTB groups (fold change > 1.2 or < 0.83, *p* < 0.05) (Fig. 4B). Elevated and diminished metabolites were depicted by red and blue colors respectively. The enriched metabolite sets clustering analysis indicated that the differential metabolites could be roughly categorized into several classes (Fig. 4C), namely glycerophosphocholines, amino acids and peptides, glycerophosphoethanolamines, polyenes, and so on. Moreover, the enriched metabolic pathway clustering analysis demonstrated that differential metabolites may be related to the urea cycle, biotin metabolism, and alanine metabolism (Fig. 4D). IPA of differentially expressed metabolites revealed several pathways, including STAT3, RhoA, mTOR, NK-κB, and TGF-β signaling pathways associated with PTB (Fig. 4E). Red and green nodes represent the upregulated and downregulated metabolites, respectively. The experiments showed that the metabolites between the two groups were vastly different.

### Identification and screening of differentially expressed metabolites

**Fig. 5:**
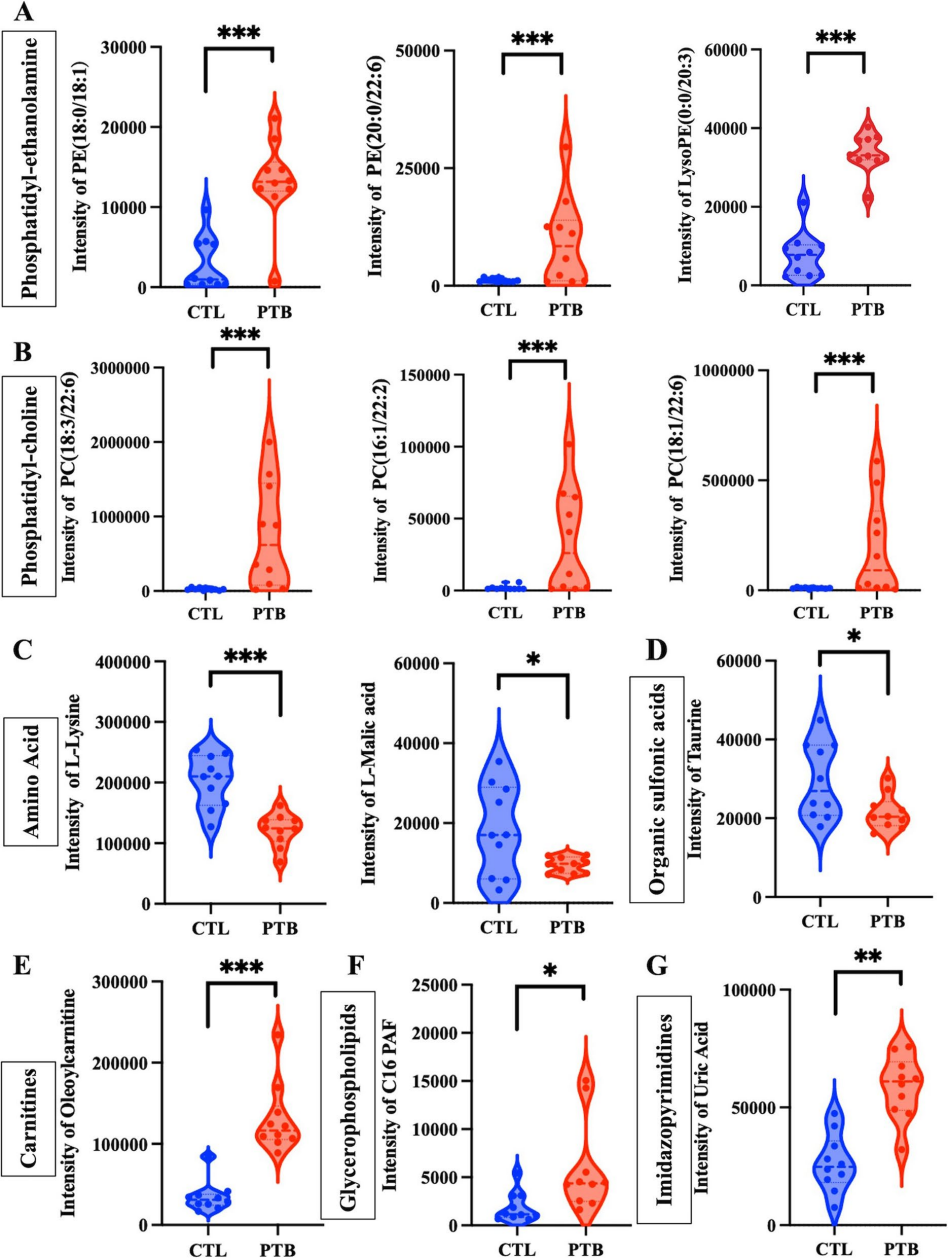
Intensity of significantly differential expressed metabolites between CTL and PTB groups. **A-B** Phosphatidylethanolamine and phosphatidylcholine were increased in PTB groups. **C** Amino acids were enriched in the CTL group. D-G. The PTB group had higher levels of organic sulfonic acids, carnitines, glycerophospholipids, and imidazopyrimidines (*p < 0.05, ** *p* < 0.01, and *** *p* < 0.001)

A total of 181 differential metabolites were identified in the PTB group (VIP > 1, *p* < 0.05), among which 96 metabolites were significantly upregulated and 85 were significantly downregulated. Among them, levels of phosphatidylethanolamine, phosphatidylcholine, organic sulfonic acids, carnitines, glycerophospholipids, and imidazopyrimidines were increased in the PTB group. On the other hand, amino acids were enriched in the CTL group (Fig. 5A-G). The most significantly changed metabolites are listed in the Table 2. Referring to Liang’s article that focused on the gestational week and peripheral blood metabolic profile [21], C16 platelet-activating factor (PAF), oleoylcarnitine, PE(18:0/18:1), uric acid, L-Lysine, L-Malic acid, and taurine were selected as candidate metabolites to explore their potential biological functions.

**Table 2:** Metabolites in the plasma of PTB mice

Class	**Name**	**VIP**	***P*-value**	**Fold change**
Phosphatidyl-ethanolamine	PE(18:0/18:1)	1.40871	0.00006733	4.45247384
Phosphatidyl-ethanolamine	PE(20:0/22:6)	1.20732	0.01144547	8.11760291
Phosphatidyl-ethanolamine	LysoPE(0:0/20:3)	1.69718	0.00000000	4.30431398
Phosphatidylcholine	PC(18:3/22:6)	1.31718	0.00451134	30.77170529
Phosphatidylcholine	PC(16:1/22:2)	1.23289	0.01000222	18.88476176
Phosphatidylcholine	PC(18:1/22:6)	1.15825	0.01818971	18.54819926
Amino Acid	Taurine	1.04184	0.02623258	0.72756712
Amino Acid	L-Lysine	1.62264	0.00008174	0.60103406
Amino Acid	L-Malic acid	1.04307	0.02522965	0.51978576
Carnitines	Oleoylcarnitine	1.75789	0.00000426	3.74689553
Glycerophospholipids	C16 PAF	1.06412	0.02201251	3.09047059
Imidazopyrimidines	Uric Acid	1.46361	0.00002286	2.22095885

### Potential biological functions of metabolites on human CSMC

**Fig. 6:**
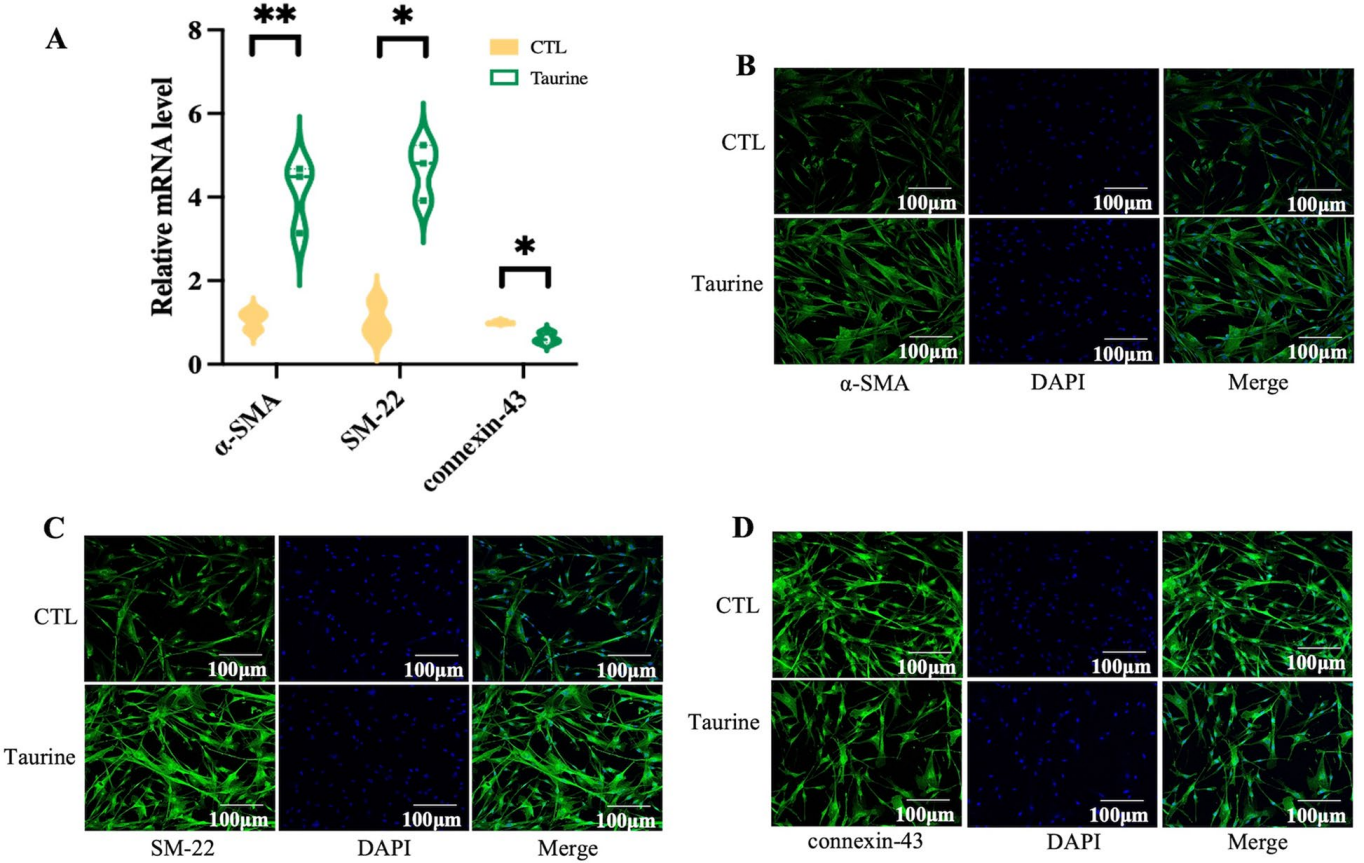
Effect of taurine on human CSMC. **A** After being treated with taurine, the level of α-SMA, SM-22 mRNA was elevated in human cervical smooth muscle cells, while the level of connexin-43 mRNA was decreased. (*p < 0.05 and ** *p* < 0.01). **B** α-SMA, SM-22, and connexin-43 expression in the human CSMC were evaluated using immunofluorescence staining. The images show that taurine enhanced the expression of α-SMA, SM-22 and weakened that of connexin-43 in the human CSMC. The cell nuclei were stained with DAPI

To further explore the biological roles of metabolites, candidate metabolites were detected. Human CSMC were treated with C16 PAF, oleoylcarnitine, PE (18:0/18:1), uric acid, L-Lysine, L-Malic acid, and taurine, separately. Only taurine was found to have an effect on human CSMC. Moreover, qPCR results showed that taurine significantly increased the levels of α-SMA mRNA and SM-22 mRNA, whereas the level of connexin-43 mRNA was decreased (Fig. 6A). Likewise, immunofluorescence staining demonstrated that taurine enhanced the expression of α-SMA and SM-22 while weakening the expression of connexin-43 in human CSMC (Fig. 6B-D).

## Discussion

PTB is the leading cause of infant mortality and morbidity. There are more than 6.6 million preterm births each year in India, China, Nigeria, Bangladesh, and Indonesia alone [22]. However, the pathogenesis of preterm birth is complicated [23]. Moreover, current interventions to prevent prematurity are largely ineffective and accompanied by undesirable side effects. Premature cervical maturation is defined as the inability of the cervix to maintain pregnancy before reaching full term, which results in progressive and painless cervical shortening, dilatation, flattening, and a funnel-shaped cervix [24]. Understanding this process may provide critical clues for PTB. Since further research about PTB is warranted to prevent and treat PTB, we evaluated the cervical changes, identified metabonomics profiles and differentially expressed metabolites in order to explore the mechanism of premature cervical ripenings in PTB.

The continual contraction and relaxation of the CSMC plays an active role in cervical remodeling and is critical in maintaining a pregnancy and is critical to maintaining pregnancy [25]. Before parturition, alterations in the contractile activity of CSMC and cervical collagen contribute to cervical maturation [26]. α-SMA, SM-22, and calponin are mature smooth muscle markers [27]. Calponin, as an inhibitor of actin activated myosin ATPase in smooth muscle cells, is an actin filament-related regulatory protein. During PTB, uterine contraction may lead to the destruction of myometrial smooth muscle cells and CSMC, subsequently causing an increase in maternal serum α-SMA, SM-22, and calponin [28]. COX-2, on the other hand, is the key limiting enzyme for the conversion of arachidonic acid into prostaglandins and is essential for regulating matrix metalloprotein expression in the breakdown of the extracellular collagen during the cervical maturation process in vivo. The elevated expression of COX-2 exhibited in the cervix may be one of the causes of early labor onset [29]. Oxytocin is a labor inducer that stimulates prostaglandin release [25]. Connexin-43 participates in growth, differentiation, and tissue remodeling, as well as coordinating cervical ripening before delivery [30, 31]. Herein, the CTL mice carried a significant amount of α-SMA, SM-22, and calponin, while OR was higher at both mRNA and protein levels in the PTB mice. Our results are consistent with previous research, demonstrating that not only the number of CSMC changed, but the expression of contraction-associated proteins was also affected in PTB. Undoubtedly, pathophysiological changes in the cervix play a vital role in inflammation-induced PTB.

With the rapid development of metabonomics technology, the function of metabolites has been identified [32]. It was already established that metabolites, as indicators, may enable PTB identification. A systematic review of the literature reported that in the study of metabonomics about PTB, 46.2% of the samples were maternal blood (serum or plasma), 31.0% were amniotic fluid, 19.2% were cervical/vaginal secretions, and 1.2% were urine [33]. Morillon et al. discovered that 20 glycerophospholipids, 12 phosphatidylcholines, 7 phosphatidylethanolamines, 1 phosphatidylinositol, 2 ceramides, and 4 sphingomyelines displayed lower levels in the plasma of PTB patients compared to controls [34]. Phospholipid is associated with biological membrane stability and inflammatory reaction and has previously been implicated in pregnancy-related complications, including PTB [35]. The researchers retrieved blood samples weekly until postpartum for non-targeted metabolome analysis. A total of 176 metabolites were identified, and 5 highly predictive compounds were screened to construct a linear prediction model. The result could accurately predict labor time and gestational week within 2 weeks [21].

To further explore the potential biological functions of these metabolites, an untargeted metabonomics analysis using LPS-induced PTB mice was executed. Herein, levels of phosphatidylethanolamine (PE), phosphatidylcholine (PC), amino acid, carnitines, and glycerophospholipid were significantly altered in the inflammation-induced PTB mice. PE is considered as a sign of cellular apoptosis [36], while PC is a constitutive plasma membrane lipid and the major transport form of DHA in plasma [37]. The imbalance of amino acids in the fetoplacental system in PTB is accompanied by a change in the production of their low molecular weight derivatives gas transmitters (NO, CO) [38]. Previous studies have reported on amino acids and acylcarnitines being potential markers of gestational age [39]. Glycerophospholipid metabolism is involved in the storage and breakdown of lipid molecules for energy, apoptosis, inflammation, and cell-membrane stabilization [34]. Taurine is a ß-aminosulfonic acid that interacts with ion channels to stabilize cell membrane and regulate cell volume. Cardiomyopathy and skeletal muscle dysfunctions are apparent in taurine deficiency [40]. Furthermore, the metabolic perturbations were related to endogenous inflammation, vascular reactivity, lipid peroxidation processes, oxidative stress, and insulin actions [41]. Even though inflammatory reactions may lead to PTB, there are no reports about the mechanism of action and effects of metabolites on PTB. Therefore, in our research, the metabonomics profile, differentially expressed metabolites and metabolic signal pathways altered in PTB mice were investigated. It was observed that the levels of phosphatidylethanolamine, phosphatidylcholine, organic sulfonic acids, carnitines, glycerophospholipids and imidazopyrimidines were increased in the PTB group. Conversely, amino acids were enriched in the CTL group. According to the differential expressed metabolites profile in mice metabolomics analysis and combined with the Liang’ research about metabolic dynamics and prediction of gestational age and time to delivery in pregnant women. Additionally, some metabolites such as C16 PAF, oleoylcarnitine, PE (18:0/18:1), uric acid, L-Lysine, L-Malic acid and taurine were selected to examine their potential biological functions. Only taurine acted on CSMC; when treated with taurine, the level of α-SMA, SM-22, and calponin were elevated. Hence, taurine may play a role in premature cervical ripening.

Altogether, this study revealed changes in the cervix and metabolomic profiles between the maternal plasma of PTB and CTL mice. Particularly, it was demonstrated that differentially expressed metabolites may induce premature cervical ripening, and the positive role of taurine in PTB was highlighted. PTB is recognized as a major health issue since its incidence has not decreased significantly over the years. So far, there is a high degree of heterogeneity in methodology and metabolite identification in the metabonomics studies about PTB. Although the predictive value of some metabolites was proven only in a small sample study, our study suggests that metabolites play a crucial role in sustaining pregnancy and inducing premature cervical ripening. Further studies are required to explore the mechanism of taurine and trace changes in its level during pregnancy. Furthermore, studying larger groups of different ethnicities will aid in confirming the relationship between PTB and taurine.

## Supplementary Information

**Additional file 1: Supplementary figure.** Morphological changes in the cervix and preterm birth. Transvaginal ultra- sonographic image of a normal cervix that was 4.79 cm long and closed at both the internal and external os (left image). The PTB cervix is characterized by cervical dilation/funneling, resulting in a Y-shaped cervix with a length of only 2.13 cm (right image).

## Data Availability

The datasets generated and/or analysed during the current study are not publicly available due we are still studying the data but are available from the corresponding author on reasonable request.

## Abbreviations

PTB: Preterm birth
CSMC: Cervical smooth muscle cells
LC–MS: Liquid chromatography-mass spectrometry
LPS: Lipopolysaccharide
H&E: Hematoxylin and eosin
α-SMA: α-Smooth muscle actin
SM-22: Smooth muscle heavy chain 22
COX-2: Cyclo-oxygenase 2
OR: Oxytocin receptor
qPCR: Quantitative polymerase chain reaction
QC: Quality control
ESI: Electrospray ionization
PCA: Principal components analysis
PLS-DA: Partial least squares discrimination analysis
VIP: Variable importance in the projection
HMDB: Human metabolome database
IPA: Ingenuity pathway analysis
KEGG: Kyoto Encyclopedia of Genes and Genomes
PAF: Platelet-activating factor
PE: Phosphatidylethanolamine
PC: Phosphatidylcholine
