# Supplementary material for: Metabonomics profile analysis in inflammation-induced preterm birth and the potential role of metabolites in regulating premature cervical ripening
Source: Reprod Biol Endocrinol. 2022 Sep 6;20:135. doi: 10.1186/s12958-022-01008-y (PMC9446521; doi:10.1186/s12958-022-01008-y)
Supplement: Supplementary file 1 — Additional file 1: Supplementary figure. Morphological changes in the cervix and preterm birth. Transvaginal ultra- sonographic image of a normal cervix that was 4.79 cm long and closed at both the internal and external os (left image). The PTB cervix is characterized by cervical dilation/funneling, resulting in a Y-shaped cervix with a length of only 2.13 cm (right image). [file 12958_2022_1008_MOESM1_ESM.docx]

Supplementary Figure


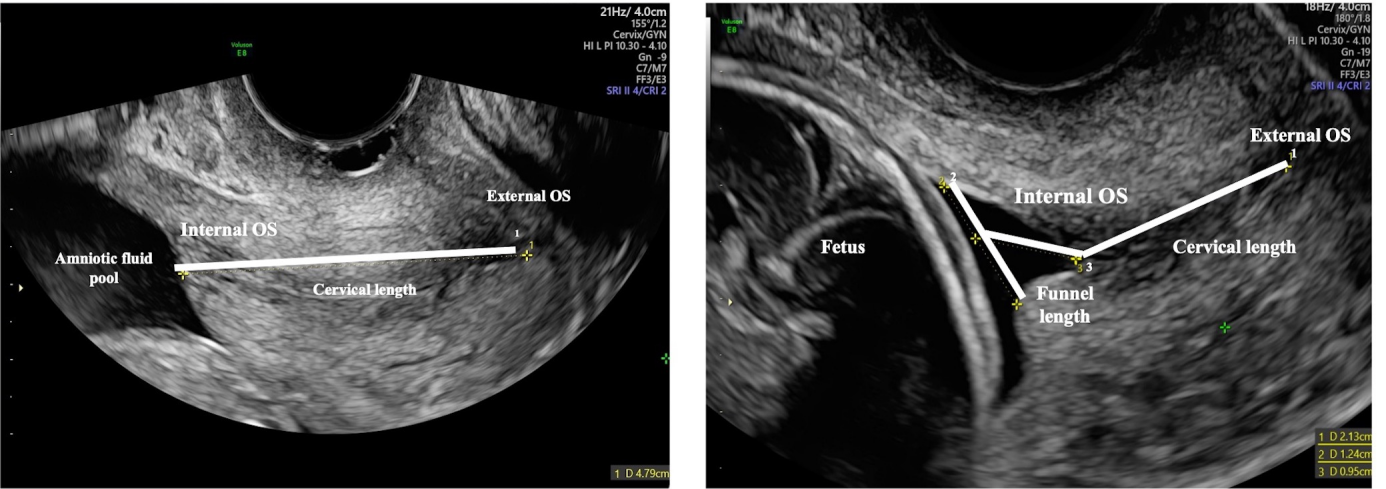


Supplementary figure **Morphological changes in the cervix and preterm birth.** Transvaginal ultra- sonographic image of a normal cervix that was 4.79 cm long and closed at both the internal and external os (left image). The PTB cervix is characterized by cervical dilation/funneling, resulting in a Y-shaped cervix with a length of only 2.13 cm (right image).
